# Superspreading of SARS-CoV-2: a systematic review and meta-analysis of event attack rates and individual transmission patterns

**DOI:** 10.1017/S0950268824000955

**Published:** 2024-10-08

**Authors:** Clifton D. McKee, Emma X. Yu, Andrés Garcia, Jules Jackson, Aybüke Koyuncu, Sophie Rose, Andrew S. Azman, Katie Lobner, Emma Sacks, Maria D. Van Kerkhove, Emily S. Gurley

**Affiliations:** 1 Department of Epidemiology, Johns Hopkins Bloomberg School of Public Health, Baltimore, MD, USA; 2 Welch Medical Library, Johns Hopkins University, Baltimore, MD, USA; 3 Department of International Health, Johns Hopkins Bloomberg School of Public Health, Baltimore, MD, USA; 4 Department of Epidemic and Pandemic Preparedness and Prevention, Emergency Preparedness Programme, World Health Organization, Geneva, Switzerland

**Keywords:** coronavirus, COVID-19, heterogeneity, infectious disease epidemiology, transmission

## Abstract

SARS-CoV-2 superspreading occurs when transmission is highly efficient and/or an individual infects many others, contributing to rapid spread. To better quantify heterogeneity in SARS-CoV-2 transmission, particularly superspreading, we performed a systematic review of transmission events with data on secondary attack rates or contact tracing of individual index cases published before September 2021 prior to the emergence of variants of concern and widespread vaccination. We reviewed 592 distinct events and 9,883 index cases from 491 papers. A meta-analysis of secondary attack rates identified substantial heterogeneity across 12 chosen event types/settings, with the highest transmission (25–35%) in co-living situations including households, nursing homes, and other congregate housing. Among index cases, 67% reported zero secondary cases and only 3% (287) infected >5 secondary cases (“superspreaders”). Index case demographic data were limited, with only 55% of individuals reporting age, sex, symptoms, real-time polymerase chain reaction (PCR) cycle threshold values, or total contacts. With the data available, we identified a higher percentage of superspreaders among symptomatic individuals, individuals aged 49–64 years, and individuals with over 100 total contacts. Addressing gaps in the literature regarding transmission events and contact tracing is needed to properly explain the heterogeneity in transmission and facilitate control efforts for SARS-CoV-2 and other infections.

## Introduction

Following the emergence of SARS-CoV-2 in 2019, the virus spread worldwide, resulting in the coronavirus disease (COVID-19) pandemic [1]. Understanding the drivers of SARS-CoV-2 transmission was crucial for formulating control measures, especially prior to the development of vaccines. Heterogeneity in transmission, particularly superspreading, was investigated early on because of its ability to cause large outbreaks [2–4]. Superspreading involves two distinct but non-mutually exclusive phenomena: a setting where many people become infected due to an environment conducive to transmission (e.g., crowded indoor settings) and individuals who are outliers in the number of secondary cases they infect due to high-risk behaviours and/or biological heterogeneity in infectiousness [5, 6]. Superspreading has been observed in several other viral infections, including SARS-CoV, MERS-CoV, Nipah, Ebola, and measles [7–12]. With SARS-CoV-2, both forms of superspreading garnered considerable attention in the literature. For example, over 140 individuals were infected during a Christmas event in Belgium in December 2020, causing over 26 deaths [13]. Likewise, one individual infected dozens of people during a choir practice in Washington, USA, in March 2020 [14].

Because superspreading events contributed substantially to local and global SARS-CoV-2 transmission [15], public health interventions were enacted to reduce their risk of occurrence. These interventions included school closures, limitations on indoor gatherings, and restrictions on visiting hospitalized patients or long-term care facilities. Many of these policies were based on limited data from the early stages of the pandemic. Moreover, published reviews and modelling of SARS-CoV-2 superspreading from this period were limited in scope and did little to disaggregate this phenomenon into distinct contributions of environment and individual characteristics. For example, studies of setting-specific transmission rates have focussed on household and healthcare transmission or geographic and temporal trends [2, 16–19] but did not address transmission heterogeneity across other social settings. Previous meta-analyses of individual-level superspreading included only a small number of papers (<26) that calculated overdispersion in transmission, missing the majority of published transmission trees and capturing data primarily from Asia [7, 8]. Early investigations of individual-level characteristics related to superspreading were also limited by incomplete contact tracing [20, 21] and a focus on clinical over demographic characteristics [20]. A more complete summary of superspreading is needed to understand the scale of transmission heterogeneity across settings and identify causes of individual heterogeneity.

The objective of this review was to summarize global heterogeneity in SARS-CoV-2 transmission events prior to widespread vaccination and the role of environmental and individual factors in superspreading. Specifically, this review aimed to identify 1) the amount of variation in attack rates across studies and events, 2) which settings had the highest attack rates, 3) the individual offspring distribution for SARS-CoV-2, and 4) the characteristics of superspreading individuals.

## Methods

### Literature search and data extraction

We conducted this systematic review and meta-analysis according to the Preferred Reporting Items for Systematic Reviews and Meta-Analyses 2020 statement [22]; see Appendix 1 for the PRISMA checklist. We included all studies of SARS-CoV-2 in humans that contained data on 1) transmission chains; 2) numbers of index cases, contacts, and infected contacts; 3) numbers of index cases and infected contacts; or 4) secondary attack rates. A clinical informationist searched PubMed, the WHO COVID database, the I Love Evidence COVID database, and Embase on 9 September 2021. No restrictions on language or start date were applied. Results were imported into EndNote X9 (Clarivate, London, UK) where duplicates were removed. Team members screened titles and abstracts and performed full-text review in Covidence (Veritas Health Innovation, Melbourne, Australia).

We extracted data using a pre-designed, study-specific spreadsheet, collecting information on paper metadata and target variables for two outcomes: transmission events and individual index cases (Table 1). Events were defined as discrete transmission settings where secondary attack rates for defined groups of people could be calculated as the number of infected cases divided by the number of exposed individuals. This definition of secondary attack rates includes both clinical and subclinical infections in some studies. Due to limited details published in the literature, we did not attempt to distinguish events associated with individual transmission chains from a single source (potentially with sequencing data) from events that aggregated multiple transmission chains together. In lieu of this distinction, we separated events into different settings and by duration of event (i.e., exposure window, in days) reported in each paper. Twelve event types were chosen to classify each event/setting described in a paper (Table 2). To describe individual contributions to transmission, we extracted data on index cases for whom contacts were followed to identify secondary transmission. We only entered data from papers where the methods were clear that contact tracing was done for at least 1 week to capture secondary transmission from index cases. For studies that did not report SARS-CoV-2 variants, we imputed the dominant variant from CoVariants data for the country and time period of interest [23]. See the Supplementary Material for additional details on the identification of papers, data extraction (Supplementary Tables S2 and S3), and bias assessment.

**Table 1.** Description of variables extracted from papers in the systematic review of SARS-CoV-2 superspreading from December 2019 to July 2021

All papers	Title Author(s) Publication year, volume, and issue Journal Study location(s) Country Administrative unit(s): state/province, county, city General study time period (e.g., start and end year/month of data collection) Diagnostic testing method (PCR, serology, rapid antigen tests, symptom diagnosis, or mixed) Variant name Any reported prevention measures implemented in the event (e.g., masking, social distancing) Number of exposed people with reported demographic characteristics (age, sex) and vaccination status
Events	Type of event/setting (e.g., nursing home residents, household transmission study, or school) Start and end date of event
Index case	Demographic characteristics (age, sex, occupation) Age group: ≤4 years, 5–12 years, 13–18 years, 19–24 years, 25–48 years, 49–64 years, ≥65 years Symptom onset (if applicable) and diagnosis dates Symptoms (text descriptions or presence/absence) Real-time PCR cycle threshold (Ct) value Specimen type Clinical outcome (if applicable) Setting of contact (e.g., work, social, and school)

**Table 2.** Types of SARS-CoV-2 secondary transmission events occurring between December 2019 and August 2021 reported in the literature

Event type	Description of outbreak location	*N*	Minimum and maximum secondary attack rate
1	Cruise ship or other densely populated watercraft (e.g., fishing vessel, aircraft carrier)	16	0.02, 1
2	Transport mode other than ships (e.g., airplane, train, car)	20	0, 0.4
3	Households, defined as co-living individuals or close contacts who always meet each other but possibly not living together (e.g., couples in romantic relationship)	115	0, 1
4	Hospital or healthcare facility, including patients, healthcare workers, and nursing home workers (if worker data were provided separately from nursing home residents)	89	0, 0.46
5	Workplace (e.g., office), including correctional officers and teachers and staff at schools	51	0, 0.86
6	School (data on students only)	32	0, 0.54
7	Public social venue (e.g., bar, concert, sporting event)	39	0, 1
8	Private social event with members of multiple households (e.g., dinner with neighbours or extended family)	12	0.01, 0.81
9	Shopping (activities in shops, markets, and department stores)	2	0, 0
10	Nursing home or long-term care facility (residents only or residents and healthcare workers if not described separately in the paper)	41	0, 0.84
11	Congregate housing other than nursing home or long-term care facility (e.g., homeless shelter, prison, summer camp)	84	0, 1
12	Mixed locations, included any combination of the above but not described separately in the paper	91	0, 1

Heterogeneity across event types was assessed based on the variance and interquartile range of secondary attack rates. Outlier events were identified for each event type as events that exceeded the estimated upper confidence interval of the meta-analysis estimated SAR for that event type or were greater than 50%.

### Statistical analyses

To characterize the type and quality of information that we were able to extract about transmission events, we performed a descriptive analysis of event data including the number of each chosen event type, starting year of the data, focal countries, diagnostic methods, event duration, and level of missingness for all variables. Because not all individuals potentially exposed during an event were tested in each study, secondary attack rates for individual events were calculated separately using the number of exposed individuals or the number tested. If either of these quantities were missing, the value was imputed based on the value present (i.e., assuming the number tested was equal to the number exposed or vice versa). The sensitivity of results to this choice of denominator was assessed in the meta-analysis of events (see the Supplementary Material).

To describe the amount of variation in attack rates across studies and events and to identify which settings had the highest SARS-CoV-2 attack rates, a meta-analysis was performed on secondary attack rates across event types using the *metafor* package in R v4.2.2 [24]. We converted secondary attack rates for each event to Freeman-Tukey double arcsine transformed proportions [25] and calculated the sampling variance. We fit a hierarchical model with a nested random effect for events within the study and no fixed effects to assess the heterogeneity in secondary attack rates attributable to these factors using restricted maximum likelihood. We calculated *I^2^*, the percentage of variance attributable to true heterogeneity, for each random effect [26] and used Cochran’s *Q* test to test if the estimated heterogeneity in secondary attack rates was greater than expected from the sampling error alone. We then fit additional mixed-effects models that included the same random effects, along with event type and event duration as fixed effects. Cochran’s *Q* was performed on these models to assess whether residual heterogeneity in secondary attack rates was greater than expected after accounting for sampling error and fixed effects. Fitted coefficients and 95% confidence intervals (CIs) from meta-analyses were back-transformed to proportions using the geometric mean of the tested individuals across all studies in each event type [25]. These back-transformed proportions are referred to as “meta-analysis estimated secondary attack rates” or “meta-analysis estimated mean attack rates” in the text and figures. For comparison with meta-analysis estimates, we also calculated the median secondary attack rate and interquartile range across events for each chosen event type.

To characterize the individual offspring distribution for SARS-CoV-2, the overall distribution of secondary cases generated by each identified index case was fit to a negative binomial distribution, following Lloyd-Smith et al. [11]. This distribution has two parameters, the mean number of secondary cases and the dispersion parameter *k* that controls the heterogeneity in secondary cases around the mean. A smaller *k* means more heterogeneity. We also estimated the percentile of index cases producing 80% of all secondary infections using a formula and code from Endo et al. [27].

Lastly, we aimed to identify the characteristics of superspreading individuals. Based on the availability of demographic characteristics and other features of index cases in the literature, we examined differences in distributions of secondary cases reported for index cases according to sex, presence/absence of symptoms, age, real-time PCR cycle threshold (Ct) value, and the number of contacts each index case had. Additional statistical tests compared these listed factors between “superspreaders” (index cases with >5 secondary cases, following Adam et al. [3]) and “non-superspreaders” (≤5 secondary cases): Chi-square tests to compare the proportion of women, the proportion of symptomatic cases, and proportion of adults or across age bins; Student’s *t*-tests to compare mean age and Ct value; and a Kruskal-Wallis test to compare the highly skewed distributions of total contacts among index cases. All statistical tests used α = 0.05 as the statistical significance threshold to identify whether superspreaders were overrepresented among certain demographic groups.

## Results

### Study selection

**Figure 1.**
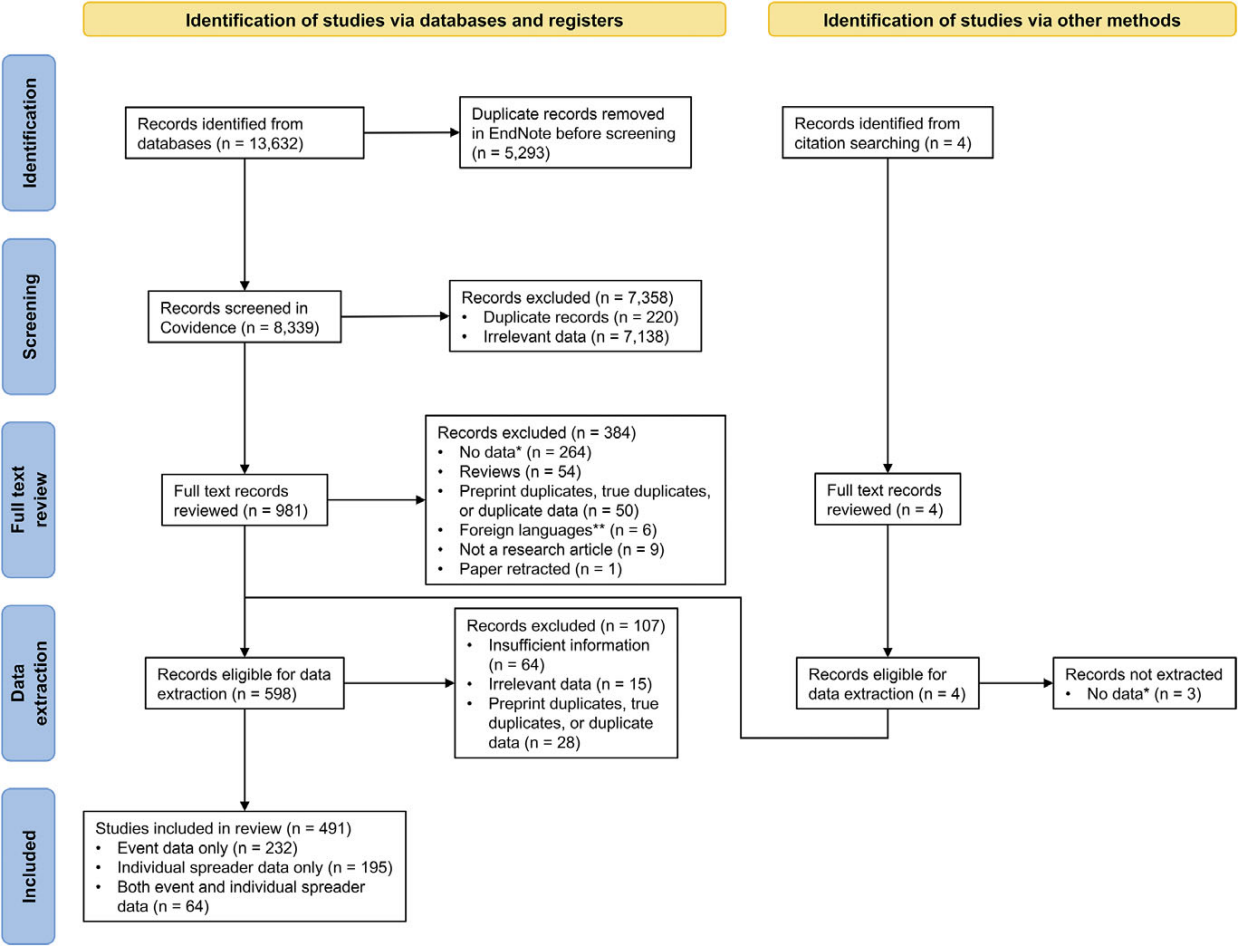
PRISMA flow diagram for the systematic review and meta-analysis of SARS-CoV-2 superspreading reported in the published literature. *There were four types of data that we sought to include: 1) transmission chain; 2) number of index cases, number of contacts, and number of infected contacts; 3) number of index cases and number of infected contacts; or 4) secondary attack rate. **Languages other than Spanish, Chinese, French, Turkish, German, and Portuguese.

We identified 13,632 articles from the four databases searched, representing 8,339 unique references (Figure 1). Of these, we excluded 7,358 records during the abstract review. For the 981 records that underwent full-text review, we excluded 384 records that were reviews or letters to the editor, contained no data on our variables of interest, or were duplicate records (preprints, true duplicates, or duplicated datasets). A total of 598 papers were assessed for eligibility for data extraction, and a further 107 papers were excluded that contained insufficient data on our outcomes of interest or were duplicates (Figure 1). We extracted data from 491 studies: 232 studies provided event data only, 195 studies provided index case data only, and 64 studies provided both data types, yielding 592 distinct events and 9,883 index cases. The 491 analysed studies were from 67 countries, with most from China (26%), the United States (17%), and South Korea (5%) (Supplementary Figure S1A). Although our search included most of 2021, nearly all data were from 2020 (94% of events, 99% of index case symptom onset or positive test dates).

### Characteristics of events

Descriptive analyses were used to characterize the type and quality of information about transmission events present in the literature. Event data were mostly from the United States (27%), China (15%), the United Kingdom (8%), and South Korea (6%) (Supplementary Figure S1B). Published papers lacked information on many variables that we aimed to extract about events (Supplementary Figure S2A). Of the 46 target data fields from articles about events, 17 had high data completeness (>80%), including those for study and event metadata, event description, time period of event (describing start and end dates of exposure), event location (country and state/province or city), and number of exposed individuals and secondary cases (Supplementary Table S3). Event durations were highly skewed, with a median duration of 34 days and an interquartile range of 13–60 days (Supplementary Figure S3). Studies used a variety of diagnostic methods to identify SARS-CoV-2 cases, though PCR was the dominant method (Supplementary Figure S4A). Other approaches included antigen tests, retrospective case identification by serology, diagnosis via symptoms or chest tomography, or a mixture of approaches. Because most studies covered events prior to the emergence of variants, most events (*N* = 532, 90%) likely involved only wild-type/ancestral SARS-CoV-2, while 14 events involved Alpha, six Beta, eight Delta, and 31 likely included a mixture of variants (e.g., during periods of variant emergence and replacement of the dominant variant).

### Heterogeneity in event secondary attack rates

**Figure 2.**
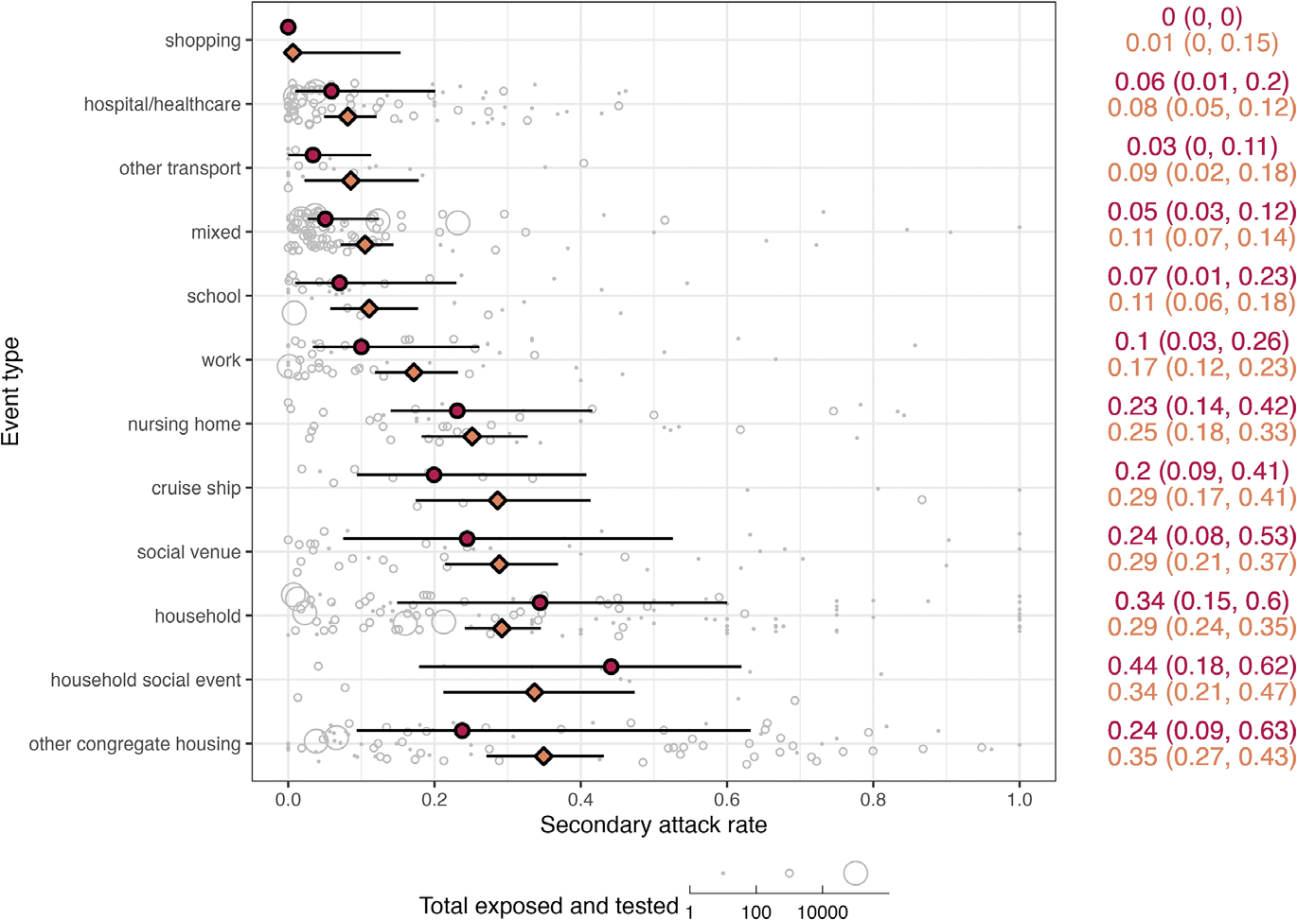
SARS-CoV-2 secondary attack rates across 12 event types occurring between December 2019 and August 2021 reported in the literature across 592 events from 296 studies. Individual event data secondary attack rates are shown as grey bubbles, varying in size according to the total number of individuals exposed and tested from the event. Median secondary attack rate for each event type is shown as red circle with a line representing the interquartile range; values are in red on the right side of the figure. Meta-analysis estimated secondary attack rate for each event type is shown as an orange diamond with a line representing the estimated 95% confidence interval; values are in orange on the right side of the figure. Event types were ranked by increasing estimated mean secondary attack rate along the left axis.

Meta-analysis of secondary attack rates was performed to describe variation in attack rates across studies and events and to rank settings by the highest attack rates. Secondary attack rates varied substantially within and among event types (Figure 2). Interquartile ranges of attack rates were lower for transport (0–11%), hospital/healthcare (1–20%), and mixed events (3–12%), whereas congregate housing (9–63%), households (15–60%), social venues (8–53%), and cruise ships (9–41%) had higher heterogeneity, with some events reporting attack rates of 100% (Table 2). Meta-analysis of secondary attack rates including a nested random effect for events within the study detected significant heterogeneity in secondary attack rates (*I^2^* = 99%, Cochran’s *Q*_*E*,591_ = 141,765, *P* < 0.0001). The random effect in the study accounted for most of the heterogeneity (*I^2^_study_* = 58%), followed by event nested within the study (*I^2^_event_* = 41%). Addition of a fixed effect for event type indicated that secondary attack rates varied significantly across event types (Cochran’s *Q*_*M*,11_ = 122, *P* < 0.0001). Meta-analysis estimated mean attack rates were lowest for shopping (0%), hospitals and healthcare (6%), transportation other than cruise ships (9%), and schools (11%) (Figure 2). Comparatively, estimated mean attack rates were 2–3 times higher (25–35%) in nursing homes, cruise ships, households, and other congregate housing settings (e.g., homeless shelters and prisons). Models including event duration and an interaction term between event type and event duration as additional fixed effects found similar levels of heterogeneity (Cochran’s *Q*_*M*,23_ = 135, *P* < 0.0001) and identified a common trend of decreasing attack rates with longer event durations across different event types, except for cruise ships and shopping (Supplementary Figure S5).

### Characteristics of individual index cases

Descriptive analyses were also used to characterize the type and quality of information about index cases found in published studies. Index case data with offspring distributions overwhelmingly came from China (36%) and India (35%) (Supplementary Figure S1C). Index case data exhibited higher missingness compared to events (Supplementary Figure S2B): of the 74 data fields extracted for index cases, the highest completeness (>60%) was seen for study and index case numbers, location (country and state/province or city), number of contacts infected, method of testing for the index case and contacts, and SARS-CoV-2 variant (Supplementary Table S4). We identified five key characteristics of index cases that could be related to superspreading, though most of these were also missing from studies: 46% of cases included data on age, 48% on sex, 10% on presence/absence of symptoms, 6% on number of contacts, and only 2% had Ct values reported. A total of 5,437 index cases (55%) contained data on at least one of these five variables. Diagnostic methods for the identification of index cases and their associated secondary cases were only reported in 61% of cases, with PCR as the primary approach (Supplementary Figure S4B,C). Most index cases (*N* = 8,565, 87%) were assumed to be infected with wild-type SARS-CoV-2 based on the location and timing of the study or test confirmation date. A mixture of variants was likely in 1,282 cases (13%), while one index case was reported with Alpha, two Beta, 11 Delta, and 22 Epsilon.

### Heterogeneity in transmission across individual index cases

**Figure 3.**
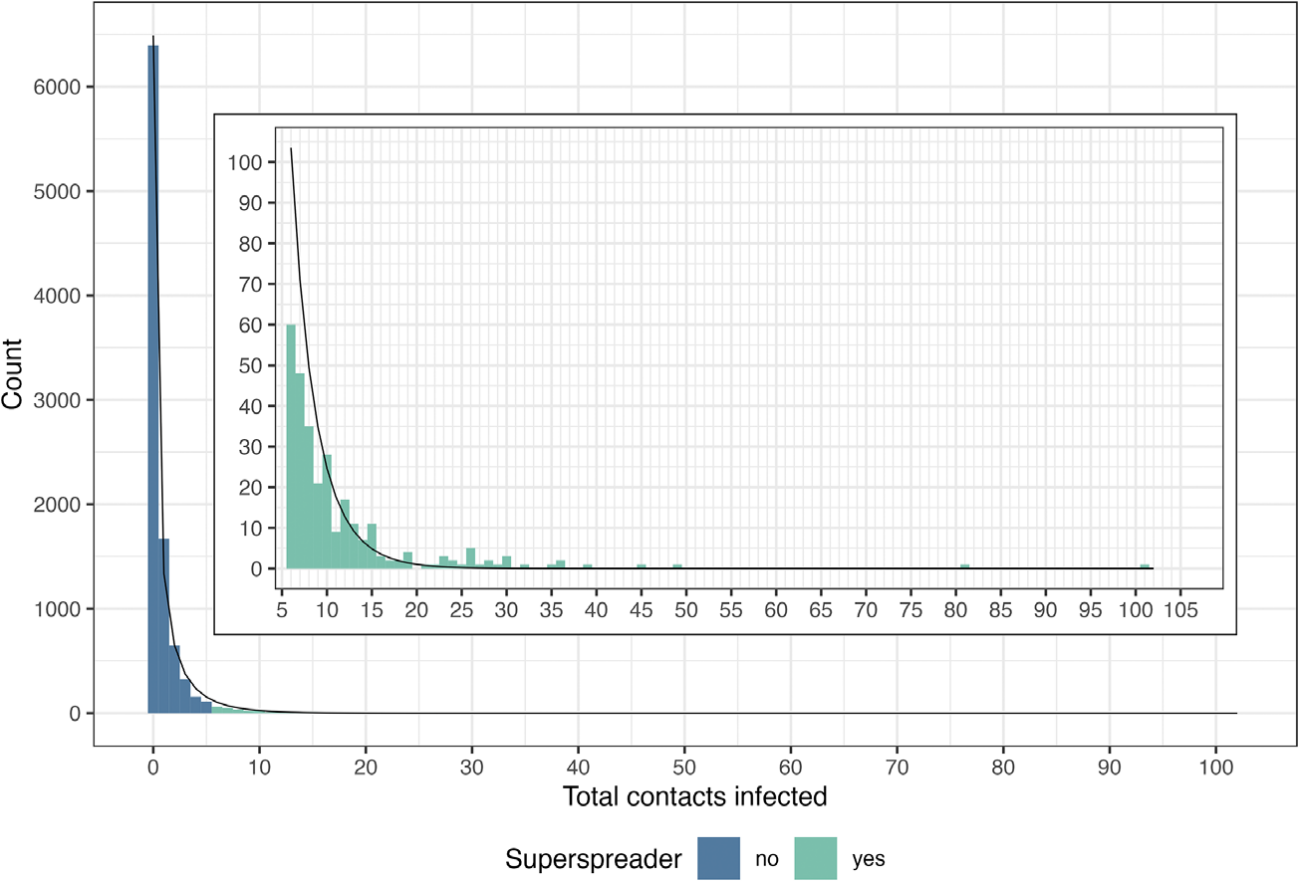
Distribution of secondary contacts infected by individual index cases (*N* = 9,591) for SARS-CoV-2 cases occurring between December 2019 and July 2021 reported in 259 studies. The black line shows the fit of the distribution to the expected negative binomial distribution. The inset shows a portion of the same data to highlight the distribution of superspreaders (index cases with >5 secondary cases).

A third goal of this analysis was to describe the offspring distribution for SARS-CoV-2 based on reported index cases. Most index cases (67%) did not transmit SARS-CoV-2 to another person and 17% transmitted to only one other individual (Figure 3). There were 287 “superspreaders” with >5 contacts infected, representing 3% of index cases. The distribution of secondary infections fit a negative binomial distribution with a mean of 0.88 (CI: 0.84–0.92) and a dispersion parameter *k* of 0.27 (CI: 0.25–0.28). Using the formula from Endo et al. [27] and the estimated mean and *k* for the negative binomial distribution, the top 17% most infectious index cases would be expected to generate 80% of all secondary cases.

### Qualities of superspreaders

**Figure 4.**
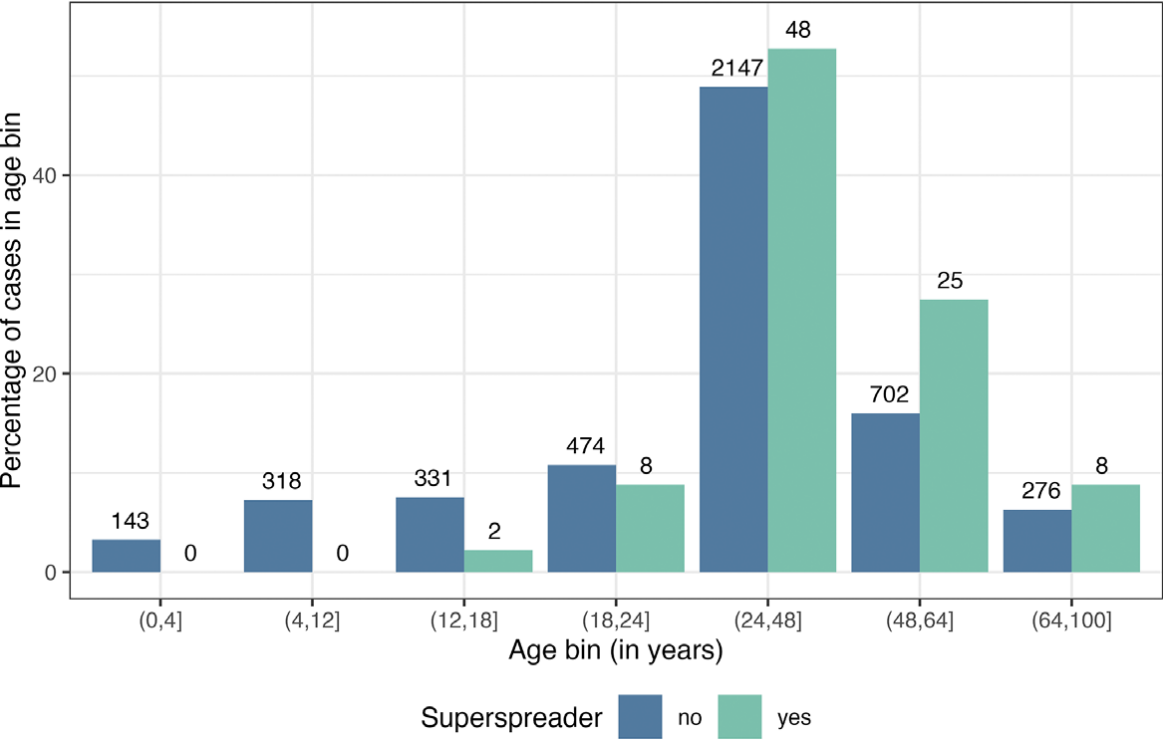
Comparison of the age distribution of superspreading index cases. The bars show the percentage of individuals within an age bin across superspreaders (index cases with >5 secondary cases) and non-superspreaders. Numbers above the bars display the raw totals and percentages are shown in Table 3.

Finally, our analysis sought to identify qualities of index cases that were associated with being a superspreader (with >5 secondary cases) compared to non-superspreaders (Table 3). The proportion of index cases with reported symptoms was higher in superspreaders (89%) than non-superspreaders (76%; χ^2^_1_ = 5.4, *P* = 0.02). Superspreaders had more than two times the mean number of contacts (79) compared to non-superspreaders (36; χ^2^_1_ = 56.6, *P* < 0.0001). Adults also made up a greater proportion of superspreaders (99%) than non-superspreaders (84%; χ^2^_1_ = 14.1, *P* < 0.0001). Index cases over 25 years of age were overrepresented among superspreaders, and no superspreaders aged 12 years and under were reported (Figure 4). When age was analysed as a continuous variable, the number of contacts infected and the frequency of superspreaders increased with age, up to around 60 years of age (Supplementary Figure S6). No significant differences by sex or Ct values were observed (Table 3). However, two adult male index cases had the highest number of secondary infections, infecting 81/104 contacts and 101/300 contacts, respectively. The former was a lecturer in Tonghua, China [28], and the latter a fitness instructor in Hong Kong, China [29].

**Table 3.** Statistical comparisons of SARS-CoV-2 superspreaders to non-superspreaders based on features reported in the literature in 259 studies for cases occurring between December 2019 and July 2021

Feature of comparison	Percentage or estimated mean for non-superspreaders (total observations)	Percentage or estimated mean for superspreaders (total observations)	Statistical test results
Female	40% (*N* = 4,543)	38% (*N* = 102)	χ^2^_1_ = 0.09, *P* = 0.76
Presence of symptoms (symptomatic)	76% (*N* = 841)	89% (*N* = 70)	χ^2^_1_ = 5.4, *P* = 0.02
Age (in bins)	(*N* = 4,391)	(*N* = 91)	χ^2^_6_ = 21.7, *P* = 0.001
≤4 years	3%	0%	
5–12 years	7%	0%	
13–18 years	8%	2%	
19–24 years	11%	9%	
25–48 years	49%	53%	
49–64 years	16%	27%	
≥65 years	6%	9%	
Age (≥18 years)	84% (*N* = 4,391)	99% (*N* = 91)	χ^2^_1_ = 14.1, *P* < 0.0001
Age (in years)	34.8 (*N* = 4,391)	43.8 (*N* = 91)	*t*_94.4_ = 5.2, *P* < 0.0001
Ct value	26.7 (*N* = 140)	24.8 (*N* = 10)	*t*_10.1_ = −0.8, *P* = 0.45
Total contacts	36 (*N* = 471)	79 (*N* = 59)	χ^2^_1_ = 56.6, *P* < 0.0001

Symptomatic cases had a higher mean number of infected contacts (2.1) compared to asymptomatic cases (0.7) (Table 4). The dispersion parameter *k* was higher for symptomatic cases than asymptomatic cases (0.43 vs. 0.11), indicating a lower variance in the number of secondary cases reported for a symptomatic case. This variance is exemplified by the lower percentage of non-transmitters (44%) and higher percentage of superspreaders (9%) among symptomatic cases compared to asymptomatic cases (79% and 4%, respectively). Compared to other age groups, individuals aged 49–64 years had the highest mean number of infected contacts (1.2), lower variance (higher *k*, 0.43), and a higher percentage of superspreaders (3%). Patterns for total reported contacts were different, with a higher mean number of infected contacts (8) as well as higher variance (lower *k*, 0.28) among index cases with >100 contacts compared to individuals with fewer contacts. This was accompanied by a higher percentage of superspreaders (28%) among individuals with >100 contacts compared to individuals with 11–100 contacts (19%) or those with 0–10 contacts (2%). Considering only symptomatic adults with a known number of contacts (*N* = 129), the percentage of superspreaders was consistently smaller as the number of contacts decreased: 26% (5/19) for individuals with over 100 contacts, 24% (8/34) for those with 21–100 contacts, 8% (2/24) for those with 11–20 contacts, and 0% for those with 10 or fewer contacts (0/52).

**Table 4.** Summary statistics describing the distribution of secondary cases among individual SARS-CoV-2 index cases occurring between December 2019 and July 2021 reported in the literature across 259 studies

Data	Sample size	Percentage with 0 contacts infected	Percentage with 1–5 contacts infected	Percentage with >5 contacts infected	Maximum contacts infected	Estimated mean contacts infected (95% CI)	Estimated overdispersion, *k* (95% CI)
All rows	9,591	67%	30%	3%	101	0.88 (0.84–0.92)	0.27 (0.25–0.28)
Female	1,866	75%	22%	2%	30	0.63 (0.56–0.71)	0.18 (0.16–0.21)
Male	2,779	74%	24%	2%	101	0.76 (0.69–0.84)	0.17 (0.15–0.19)
Asymptomatic	214	79%	17%	4%	25	0.75 (0.43–1.08)	0.11 (0.07–0.16)
Symptomatic	697	44%	47%	9%	81	2.06 (1.8–2.3)	0.43 (0.36–0.49)
Age ≤4 years	143	90%	10%	0%	3	0.2 (0.07–0.32)	0.09 (0.01–0.17)
Age 5–12 years	318	97%	3%	0%	3	0.04 (0.006–0.07)	0.03 (–0.006–0.06)
Age 13–18 years	333	94%	6%	1%	26	0.23 (0.08–0.38)	0.03 (0.01–0.05)
Age 19–24 years	482	88%	10%	2%	19	0.38 (0.23–0.52)	0.06 (0.04–0.09)
Age 25–48 years	2,195	76%	22%	2%	101	0.73 (0.65–0.82)	0.15 (0.13–0.17)
Age 49–64 years	727	54%	43%	3%	35	1.16 (1.01–1.31)	0.43 (0.36–0.51)
Age ≥65 years	284	58%	39%	3%	18	1 (0.79–1.21)	0.43 (0.3–0.57)
Ct value ≤25	67	42%	52%	6%	12	1.48 (1–1.96)	0.86 (0.31–1.42)
Ct value >25	83	52%	41%	7%	26	1.45 (0.87–2.02)	0.4 (0.2–0.6)
0–10 total contacts	281	49%	48%	2%	9	1.12 (0.93–1.31)	0.83 (0.53–1.14)
11–100 total contacts	195	47%	34%	19%	39	3.1 (2.29–3.91)	0.32 (0.23–0.41)
101–1,000 total contacts	54	35%	37%	28%	101	8 (3.92–12.07)	0.28 (0.16–0.4)

## Discussion

In this study, we aimed to characterize the heterogeneity in SARS-CoV-2 transmission among different settings and across individuals reported in the literature. Regarding transmission settings, our meta-analysis identified substantial heterogeneity in attack rates across 12 chosen event types, with higher mean attack rates in nursing homes, cruise ships, households, and other congregate housing settings compared to shopping, hospitals and healthcare, other transportation, and schools. Regarding individual transmission heterogeneity, our results indicate substantial heterogeneity in transmission from individuals, as observed in other studies [30–32] and evidenced by the skewed degree distribution for index cases and the estimate of the dispersion parameter *k.* Our estimate of *k* (0.27, CI: 0.25–0.28) is within the range of previous estimates for a similar period of the pandemic, with values frequently in the range of 0.1–0.7 [3,7,8,27,33]. We found that most cases did not transmit to another person and a small proportion (3%) of individuals were superspreaders (with >5 secondary cases). While data on the demographics of index cases were not consistently reported, the data that were available indicate that superspreaders were more likely to be symptomatic than non-superspreaders, more likely to be adults (with overrepresentation in the 49–64 age group), and had more contacts.

Our ranking of event types by attack rate reinforces our existing understanding of SARS-CoV-2 that transmission is more likely in dense indoor gatherings or close and frequent contact among co-living individuals, especially in households [15]. Published meta-analyses covering the early pandemic (pre-2021) estimated pooled household secondary attack rates of 17–21% [16, 18, 19, 34, 35], with household attack rates consistently higher than those in healthcare, work, or travel settings [16, 19]. Our pooled household secondary attack rate over 115 events was 29%, higher than these earlier studies but similar to the 31% estimate from Madewell et al. [18] for studies covering July 2020 to March 2021. The higher value may be explained by the emergence of the Alpha and Delta variants and the larger second and third waves of the pandemic occurring in some countries during 2021.

The literature on SARS-CoV-2 transmission events rarely reported on the epidemiological context and characteristics of different populations exposed, which could help explain variation in attack rates. While the timing and location of events may help to explain some variation within event types, the remaining variation could depend on event duration (as shown by Supplementary Figure S5) and time spent indoors, types of activities occurring (e.g., exercise, singing) [36, 37], and the age groups present at the event. For example, the age of individuals interacting in these contexts appears to also influence propensity for transmission, as evidenced by the large difference in attack rates within schools versus nursing homes. Children and adolescents are frequently found to have lower household infection risk than working-age adults [18, 19, 21, 35] and older adults have a higher risk of infection and severe disease than younger ages [18, 35]. In studies that assessed transmission among school-age children, teachers, and their household contacts, attack rates among children at school were lower than among teachers and the household contacts of children and teachers [38, 39]. Variation in the stringency of interventions (e.g., masking requirements, physical distancing, and lockdowns) across countries and over time also could have affected attack rates across settings. As shown in Supplementary Figure S6 comparing attack rates for events in the United States and China, two locations with differing levels of implemented control measures, a meta-analysis estimated attack rates were lower across event types for China, though the largest differences between countries were observed for transmission in social venues and mixed settings. Environmental factors such as humidity, room size, ventilation, and air flow [5] could also augment transmission across settings, but these were absent in the literature.

Analysis of index case demographics also highlighted age as an important factor in SARS-CoV-2 superspreading. While age was only reported in 46% of index cases, nearly all superspreading individuals were adults and there were no reported superspreaders 12 years of age and under, consistent with other reviews [40]. Individual and age-related heterogeneity in the amount and assortative patterns of social contacts likely influence superspreading as well. Evidence supports lower transmission from children compared to adults [16, 21, 31, 34]. Remaining heterogeneity in individual infectiousness may derive from differences in genetic susceptibility [41, 42], body size (accounting for age) [43], baseline lung volume and function [32], immunocompromising disease or co-infection [44, 45], or the loudness and wetness of speech [36]. The relative importance of these characteristics to SARS-CoV-2 transmission at a population level is unknown and may be challenging to measure and report at scale. Future work on COVID-19 and other respiratory diseases should address these hypotheses.

While our systematic review is the most comprehensive assessment of SARS-CoV-2 superspreading to date, a principal limitation of our analysis was the incomplete data available in the published studies. Beyond information provided about event timing and location, very few studies reported any demographics of the exposed individuals, their COVID-19 vaccination status (once introduced), history of prior SARS-CoV-2 infection, or the density and amount of time indoors. For index cases, some studies reported demographic information and the presence/absence of symptoms, but this was atypical. We also had trouble deducing whether contact tracing was performed for all reported cases in transmission chains, especially for terminal nodes. It was not always clear whether cases did not transmit or whether data were missing due to lack of contact tracing, so these cases had to be omitted from the analysis. Testing and tracing policies differed between countries, which affected the collection of index cases that ended up in our review. For this reason, data on index cases are missing from many countries and transmission chains from some countries may be less complete than others. Similarly, the effectiveness of testing and tracing policies varies across settings (e.g., easier in households than large social gatherings), which affects the completeness of transmission chains and likely influences which outbreaks get published. There were numerous papers that we reviewed with transmission chains that were simply too incomplete or uncertain for us to extract index case data from them. However, without reporting of testing and tracing policies or the effectiveness of tracing efforts within each paper, or a comprehensive database or systematic review of this information in the literature, these remain uncertainties that must be addressed with better data.

Another limitation of this review was the variation in case detection methods across studies. Not all studies reported the number of contacts that were tested from events, and we assumed for missing cases that the number tested was the same as the number exposed. Our sensitivity analysis, using exposed contacts for all events as the denominator for attack rates instead of tested contacts, showed that estimated mean attack rates were consistently lower across event types, but the ranking of event types was relatively stable (Supplementary Figure S7). However, case definitions also varied by study. Some studies reported only symptomatic cases or only performed diagnostic tests (e.g., PCR) on symptomatic individuals, thereby missing all reporting of asymptomatic or symptomatic cases that did not meet the criteria for reporting/testing, as well as any associated secondary cases. These missing contacts may be undercounted for both the numerator (contacts that are infected but asymptomatic) and the denominator (including contacts that are asymptomatic and uninfected), which could move attack rates in either direction. Limiting testing to symptomatic contacts has a more predictable effect on individual case degree distributions, reducing the apparent proportion of individuals who transmit and the secondary cases among individuals who do transmit. Case ascertainment also likely varied by event setting, contributing additional uncertainty in estimated attack rates. For example, performing contact tracing and testing a greater number of contacts was probably easier in settings with consistent or recorded populations like households, schools, and nursing homes than in large social venues like nightclubs. Differences in estimated attack rates by event type may be less drastic than we observed if case ascertainment could be properly addressed with additional ground truth data, that is, community asymptomatic testing.

Since case detection depends partly on the presence of symptoms, some care should be taken in interpreting the finding that superspreaders were more likely to have symptoms than non-superspreaders. We performed an additional analysis on the presence of symptoms across different demographic factors reported in papers (see Supplementary Table S6). The only trend we saw was for age, where the presence of symptoms was somewhat higher for older adults (49 and older). This may have slightly skewed detection of superspreaders among older adults. However, there were still hundreds of children with symptoms reviewed as index cases, so there were ample opportunities for them to be identified as superspreaders. Therefore, we remain confident in our findings about the rarity of superspreaders among children. However, data from human challenge trials with SARS-CoV-2 have shown that individuals with the highest viral emissions did not have the most severe symptoms, but these super-emitters were also not asymptomatic [32]. These super-emitters, and the majority of superspreaders reported in the literature, tend to have mild to moderate symptoms [32, 40]. While the importance of asymptomatic transmission of SARS-CoV-2 should be acknowledged, numerous studies have shown that transmission is more likely from symptomatic individuals compared to completely asymptomatic individuals [18, 21, 46–48]. However, additional studies that overcome issues of case ascertainment should be done to assess the role of asymptomatic individuals in SARS-CoV-2 superspreading.

To improve our understanding of the drivers of heterogeneity in transmission, we propose standard and consistent reporting on transmission for all outbreaks, as feasible, including details on the epidemiological context of transmission events and complete line lists of cases following contact tracing, with information on case demographics (age, sex, and occupation), diagnosis (presence/absence of symptoms, symptom description, test date and results), duration of contact tracing, and number of contacts and demographic information for contacts (see Appendix 2). Details on the duration of contact tracing should include the entire period of case finding and how long cases were followed to detect any secondary cases. We recognize the challenge of collecting, storing, and sharing identifiable data from outbreak investigations while continuing to assure confidentiality and improve trust in the health system. However, developing such a reporting system should be a priority for public health as the information has important implications for reducing the spread of infectious pathogens.

Our comprehensive review found substantial heterogeneity in transmission of SARS-CoV-2, highlighting the settings and individual characteristics that might be most important to target for controlling superspreading. Secondary attack rates were highest in co-living situations where prolonged contact between individuals facilitated transmission, though there was substantial variation in attack rates within similar settings that remained unexplained and could be disentangled in future meta-analyses focussed on the relative influence of built environment, social setting, and control measures on transmission. Given the moderate attack rates among minors in school and the rarity of children among superspreaders, interventions targeting these age groups may be less efficient at preventing SARS-CoV-2 superspreading and could be deprioritized in favour of interventions focusing on adults [21, 49], especially those with symptoms and individuals with many daily close contacts. Acknowledging that there remain substantial gaps in data that limit our inference about superspreading, we advocate for consistent reporting on infectious disease outbreaks, ideally with detailed line lists, to facilitate knowledge synthesis about transmission patterns and superspreading in the future.

## Supporting information

McKee et al. supplementary material 1 McKee et al. supplementary material

McKee et al. supplementary material 2 McKee et al. supplementary material

McKee et al. supplementary material 3 McKee et al. supplementary material

McKee et al. supplementary material 4 McKee et al. supplementary material

## Data Availability

All the data were from publicly available databases. The complete database of extracted information from included studies is provided in Appendix 3.

## Supplementary material

The supplementary material for this article can be found at http://doi.org/10.1017/S0950268824000955.
